# Supplementary material for: NUP98 and RAE1 sustain progenitor function through HDAC-dependent chromatin targeting to escape from nucleolar localization
Source: Commun Biol. 2023 Jun 23;6:664. doi: 10.1038/s42003-023-05043-2 (PMC10290086; doi:10.1038/s42003-023-05043-2)

NUP98 and RAE1 sustain progenitor function through HDAC-dependent chromatin targeting to escape from nucleolar localization

Amy E. Neely, Laura A. Blumensaadt, Patric J. Ho, Sarah M. Lloyd, Junghun Kwoen, Ziyou Ren, Xiaomin Bao

List of Supplementary Items:

This pdf file: Supplementary Figures 1-9 and legend.

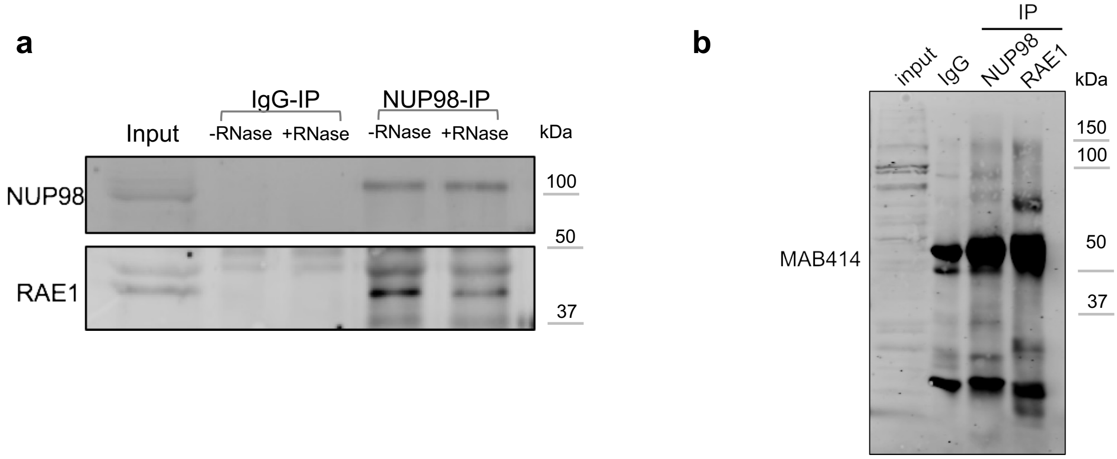

Supplementary Figure 1. Interaction between NUP98 and RAE1 in the progenitor-state keratinocytes. (a) Western blot comparing NUP98 immunoprecipitation (IP) with or without the addition of RNase, detected by NUP98 or RAE1 antibodies. (**b**) Western blot of NUP98 or RAE1 IP detected by MAB414.

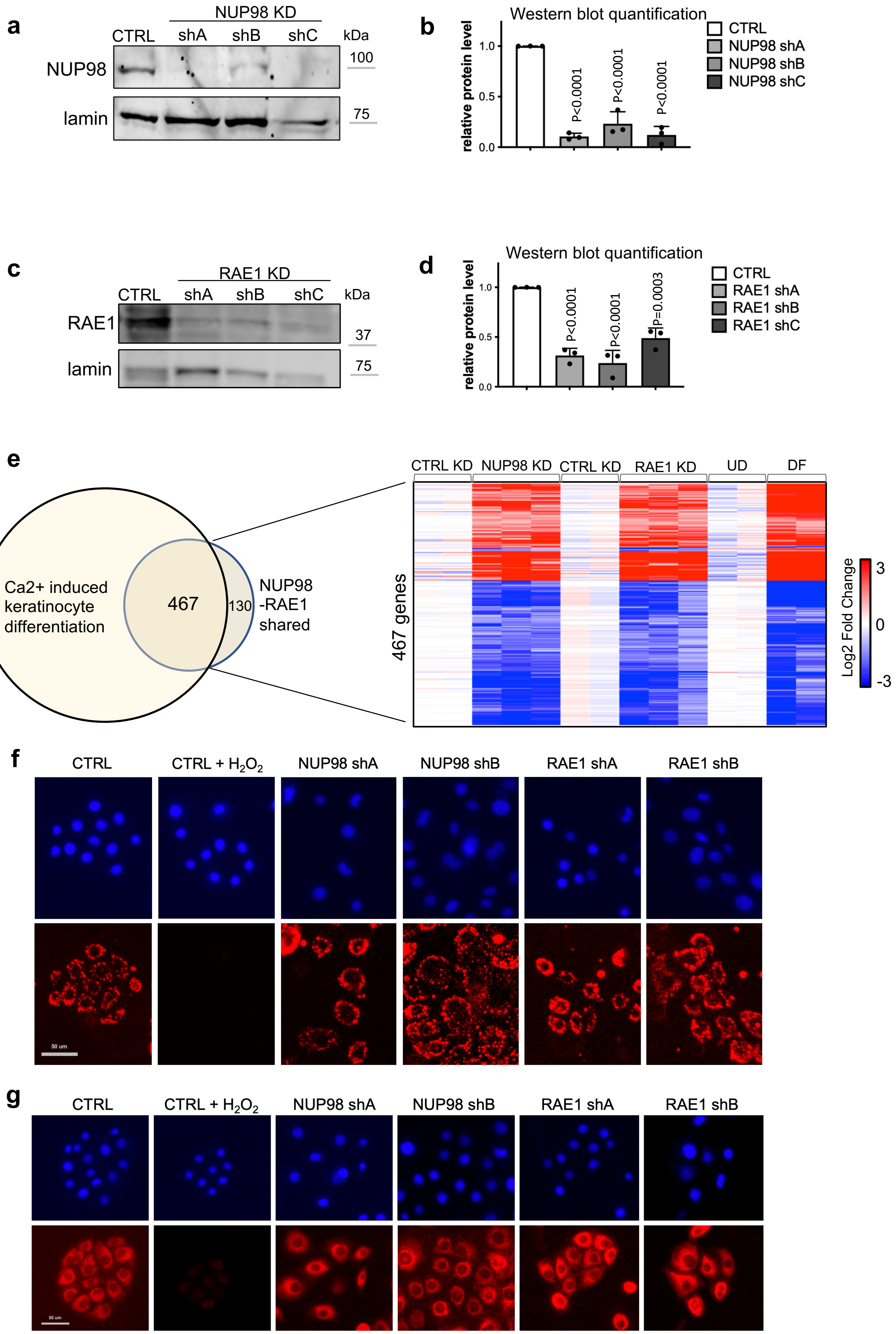
a
NUP98 KD
CTRL
shA
shB
shC
kDa
NUP98
100
lamin
75
b
Western blot quantification
relative protein level
P<0.0001
P<0.0001
P<0.0001
CTRL
NUP98 shA
NUP98 shB
NUP98 shC
c
RAE1 KD
CTRL
shA
shB
shC
kDa
RAE1
37
lamin
75
d
Western blot quantification
relative protein level
P<0.0001
P<0.0001
P=0.0003
CTRL
RAE1 shA
RAE1 shB
RAE1 shC
e
Ca2+ induced keratinocyte differentiation
467
130
NUP98 -RAE1 shared
CTRL KD
NUP98 KD
CTRL KD
RAE1 KD
UD
DF
467 genes
Log2 Fold Change
3
0
-3
f
CTRL
CTRL + H_2O_2
NUP98 shA
NUP98 shB
RAE1 shA
RAE1 shB
50 um
g
CTRL
CTRL + H_2O_2
NUP98 shA
NUP98 shB
RAE1 shA
RAE1 shB

Supplementary Figure 2. NUP98 or RAE1 knockdown impairs progenitor function. (**a,b**) Western blots and quantifications showing the knockdown efficiency of NUP98 shRNAs at the protein level, with lamin as the loading control (one-way ANOVA with post-hoc test, N=3, data are represented as mean +/- standard deviation). (**c,d**) Western blots and quantifications showing the knockdown efficiency of RAE1 shRNAs at the protein level, with lamin as the loading control (one-way ANOVA with post-hoc test, N=3, quantification data are represented as mean +/- standard deviation). (**e**) Pie chart and heatmap comparing the shared differentially expressed genes with NUP98 or RAE1 knockdown, versus the genes differentially expressed with calcium-induced keratinocyte differentiation. **(f)** Representative images of apoptosis detection using the JC-1 stain in keratinocytes with NUP98 or RAE1 knockdown as compared to non-targeting control knockdown. Control knockdown keratinocytes treated with H_2O_2 were used as positive controls for apoptosis detection (scale bar =50μm). **(g)** Representative images of apoptosis detection by the MitoView stain in keratinocytes with NUP98 or RAE1 knockdown as compared to the non-targeting control. Control knockdown keratinocytes treated with H_2O_2 were used as positive controls for apoptosis detection (scale bar=50μm).

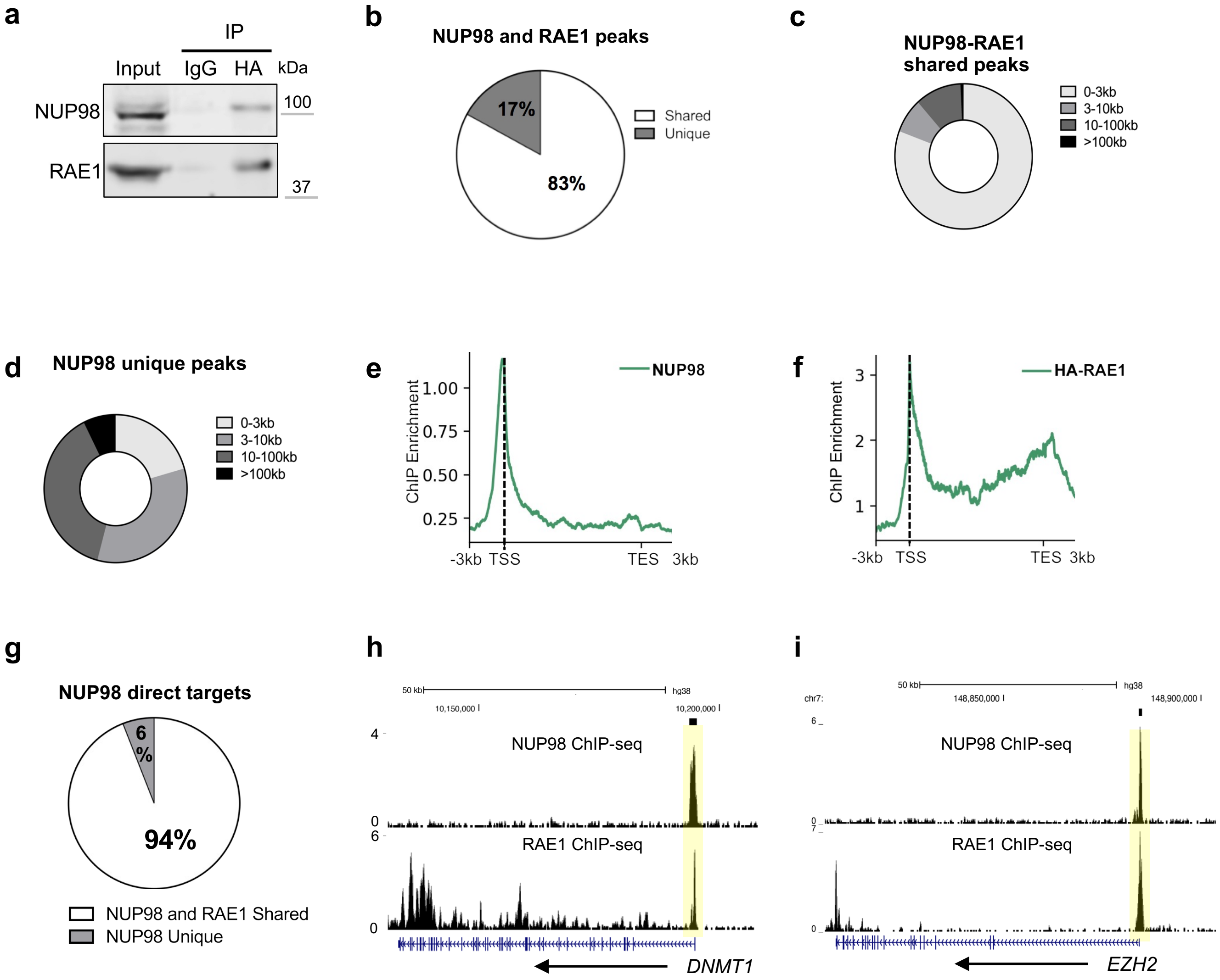

Supplementary Figure 3. RAE1 binds to chromatin together with NUP98. (**a**) Western blot images showing the co-immunoprecipitation between HA-RAE1 and NUP98. (**b**) Pie chart showing that 83% of NUP98 ChIP-seq peaks overlap with RAE1 ChIP-seq peaks. (**c,d**) Distances of NUP9-RAE1 shared peaks or NUP98-unique peaks to the nearest TSSs. The majority of NUP98-RAE1 shared peaks, but not the NUP98-unique peaks, are within 0-3kb to the nearest TSSs. **(e, f)** Average diagrams showing the enrichment of NUP98 or RAE1 near the TSSs. **(g)** Pie chart showing that 94% of the NUP98 direct target genes are also associated with RAE1 ChIP binding. (**h,i**) Representative ChIP-seq tracks showing the colocalization of NUP98 and RAE1 at the TSSs of their target genes, such as DNMT1 and EZH2.

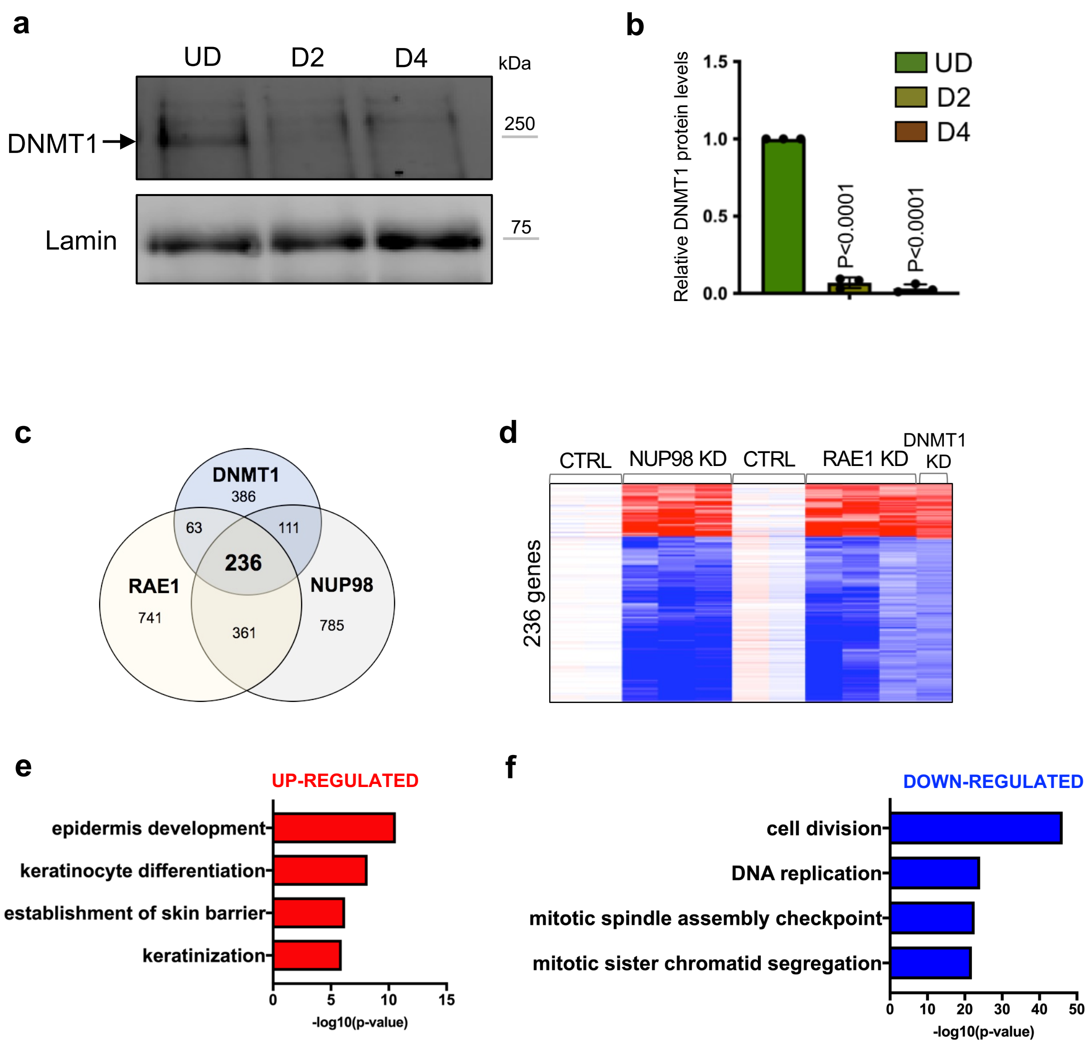

Supplementary Figure 4. DNMT1 reduction partially accounts for the differentiation induction and cell cycle inhibition in NUP98 or RAE1 knockdown. (**a**,**b**) western blots and quantifications showing the downregulation of DNMT1 in keratinocyte differentiation, with lamin as the loading control (one-way ANOVA with post-hoc test, N=3, data are represented as mean +/- standard deviation). (**c**,**d**) Venn diagram and heatmaps comparing the differentially expressed genes with NUP98, RAE1, or DNMT1 knockdown in human keratinocytes. (**e**,**f**) Top Gene Ontology terms of upregulated (red) or downregulated (blue) for the 236 genes shared among NUP98, RAE1 and DNMT1 knockdown.

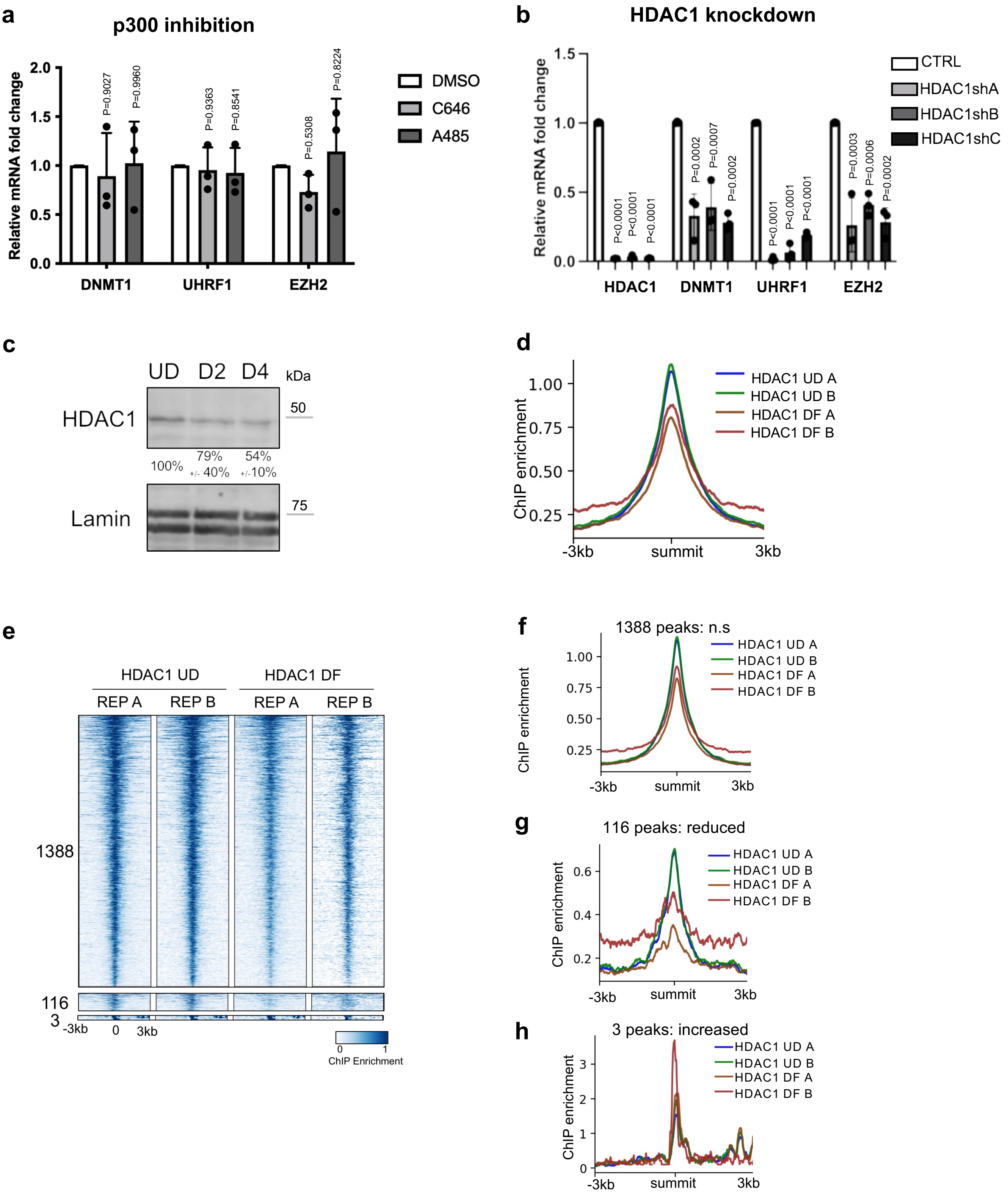

a
p300 inhibition
Relative mRNA fold change
DMSO
C646
A485
P=0.9027
P=0.9960
P=0.9363
P=0.8541
P=0.5308
P=0.8224
DNMT1
UHRF1
EZH2
b
HDAC1 knockdown
Relative mRNA fold change
CTRL
HDAC1shA
HDAC1shB
HDAC1shC
P<0.0001
P<0.0001
P<0.0001
P=0.0002
P=0.0007
P=0.0002
P<0.0001
P<0.0001
P<0.0001
P=0.0003
P=0.0006
P=0.0002
HDAC1
DNMT1
UHRF1
EZH2
c
UD
D2
D4
kDa
HDAC1
50
100%
79% +/- 40%
54% +/-10%
Lamin
75
d
ChIP enrichment
HDAC1 UD A
HDAC1 UD B
HDAC1 DF A
HDAC1 DF B
-3kb
summit
3kb
e
HDAC1 UD
HDAC1 DF
REP A
REP B
REP A
REP B
1388
116
3
-3kb
0
3kb
ChIP Enrichment
f
1388 peaks: n.s
ChIP enrichment
HDAC1 UD A
HDAC1 UD B
HDAC1 DF A
HDAC1 DF B
g
116 peaks: reduced
ChIP enrichment
HDAC1 UD A
HDAC1 UD B
HDAC1 DF A
HDAC1 DF B
h
3 peaks: increased
ChIP enrichment
HDAC1 UD A
HDAC1 UD B
HDAC1 DF A
HDAC1 DF B

Supplementary Figure 5. HDAC1, but not p300, cooperates with NUP98 and RAE in gene regulation. (**a**) qRT-PCR comparing the gene expression of representative NUP98-RAE target genes in keratinocytes treated with a p300 inhibitor (C646 or A485) or DMSO (one-way ANOVA with post-hoc test, N=3, data are represented as mean +/- standard deviation). (**b**) qRT-PCR comparing the expression of representative NUP98-RAE target genes in keratinocytes expressing an HDAC1 shRNA or non-targeting control shRNA (one-way ANOVA with post-hoc test, N=3, data are represented as mean +/- standard deviation). (**c**) Western blot and quantifications showing HDAC1 protein expression levels in keratinocyte differentiation, with lamin as the loading control (one-way ANOVA with post-hoc test, N=3, data are represented as mean +/- standard deviation). (**d**) Summit-centered average profile plot comparing HDAC1 ChIP enrichment in undifferentiated (UD) and differentiated (DF) keratinocytes. N=2. (**e-h**) heatmap and average diagrams comparing HDAC1 ChIP enrichment at NUP98 ChIP-seq peaks. The majority of these peaks (1388) don’t show drastic reduction of HDAC1 ChIP enrichment in the differentiation state. Only 116 peaks show significantly reduced HDAC1 enrichment in differentiation, and only 3 peaks show significantly increased HDAC1 enrichment in differentiation.

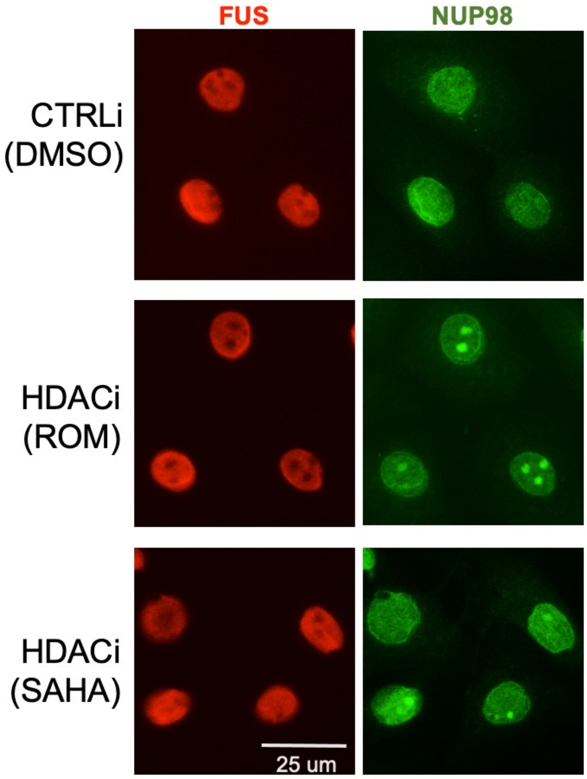

Supplementary Figure 6. HDAC-inhibition-induced NUP98 nucleolar targeting is independent of FUS. Representative images of immunofluorescence staining of NUP98 and FUS in keratinocytes treated with HDAC inhibitors or DMSO control for 24 hours (scale bar=25μm).

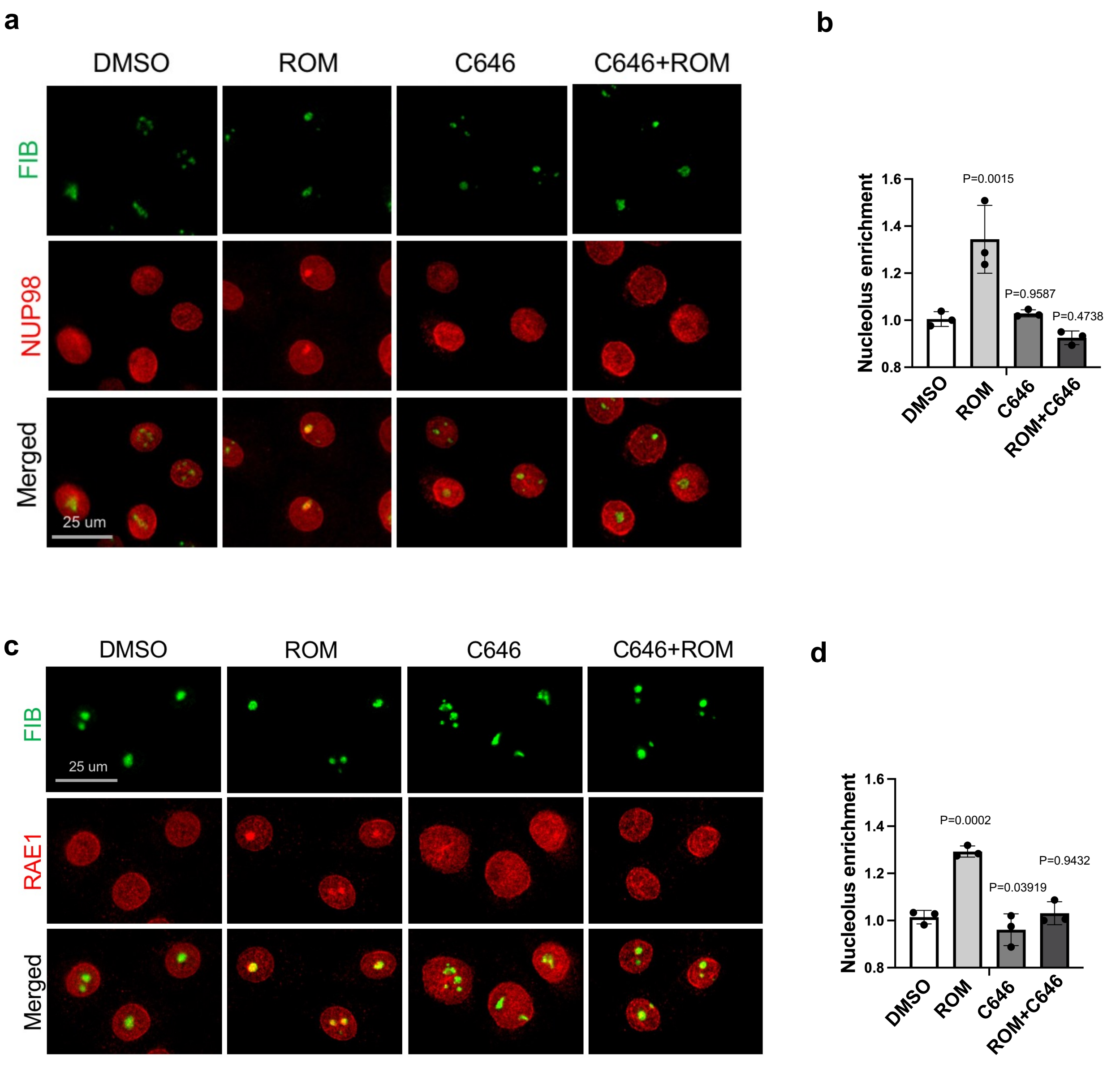

Supplementary Figure 7. HAT inhibition diminishes the nucleolar enrichment of NUP98 and RAE1 induced by HDAC inhibition. (a,b) Representative images and quantifications of NUP98's enrichment in the nucleolus in keratinocytes treated with the HAT inhibitor C464 and/or the HDAC inhibitor Romidepsin (ROM) (one-way ANOVA with post-hoc test, N=3, data are represented as mean +/- standard deviation, scale bar=25μm). (**c,d**) Representative images and quantifications of RAE1's enrichment in the nucleolus in keratinocytes treated with the HAT inhibitor C464 and/or the HDAC inhibitor Romidepsin (ROM). (one-way ANOVA with post-hoc test, N=3, data are represented as mean +/- standard deviation, scale bar=25μm).

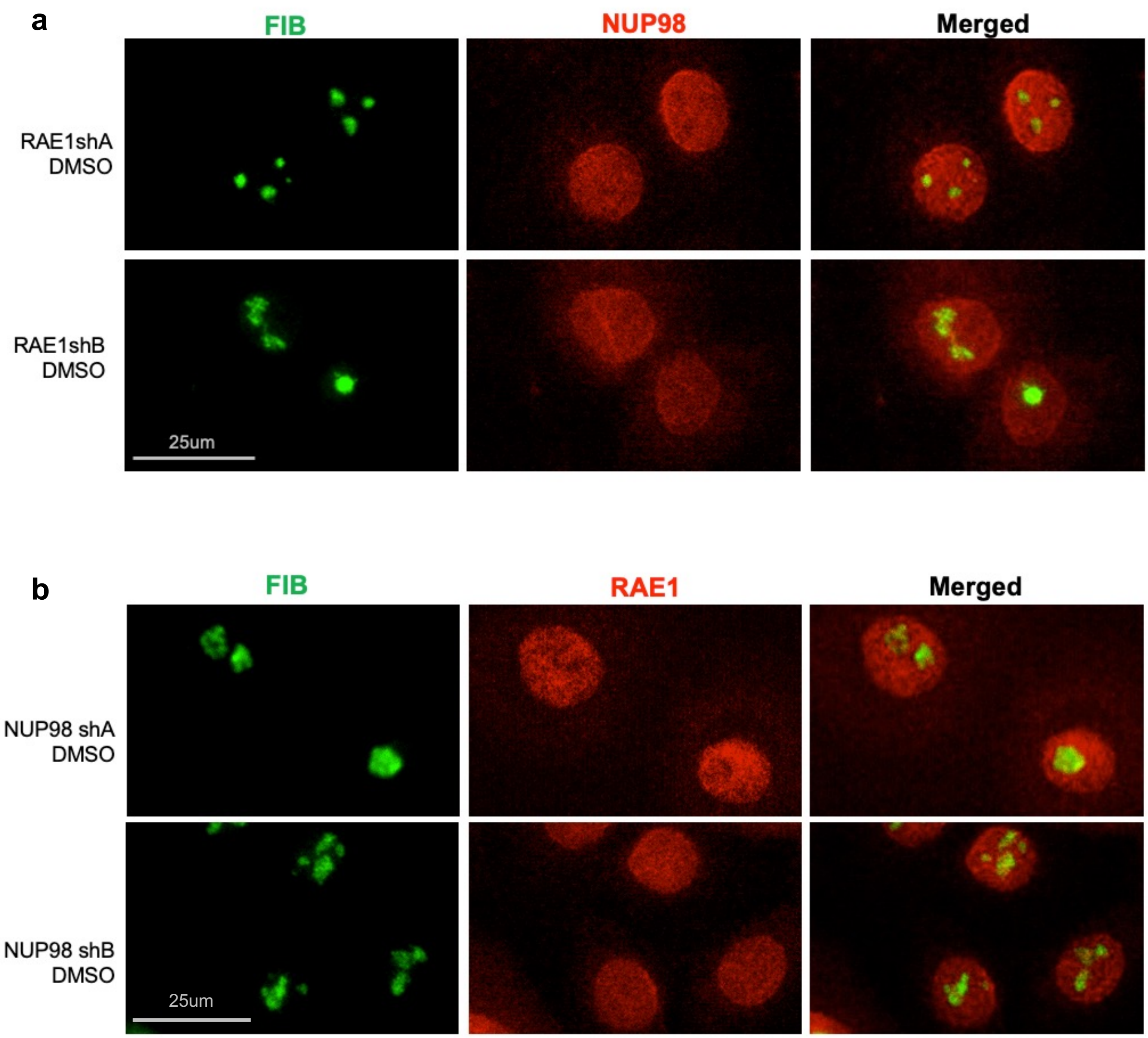

Supplementary Figure 8. NUP98 or RAE1 knockdown alone does not induce nucleolar localization without HDAC inhibition. (**a**) Representative images of immunofluorescence staining of NUP98 (red) and Fibrillarin (green) in keratinocytes with RAE1 knockdown versus control, treated with DMSO (scale bar=25μm). (**b**) Representative images of immunofluorescence staining of RAE1 (red) and Fibrillarin (green) in keratinocytes with NUP98 knockdown versus control, treated with DMSO (scale bar=25μm).

Supplementary Figure 9. uncropped westerns

Fig 1b

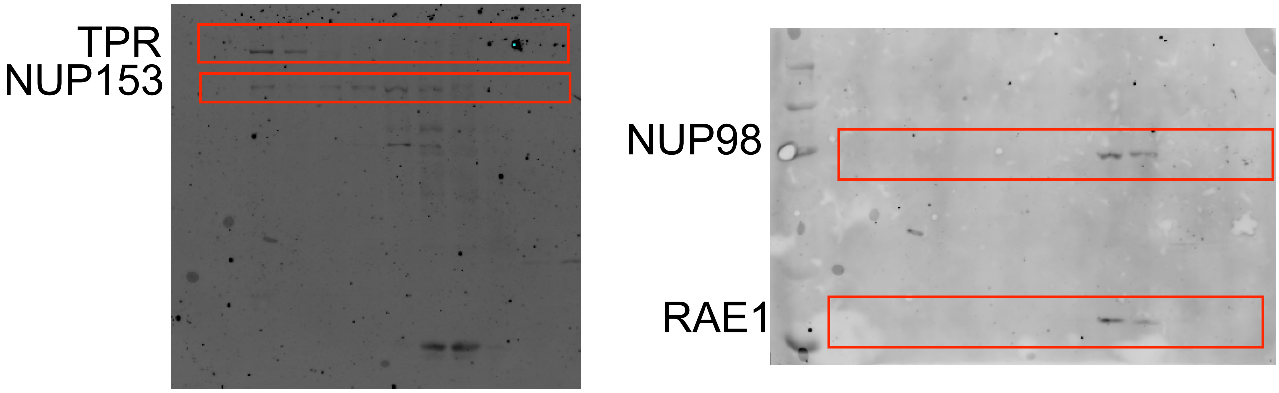

Fig 1c&d

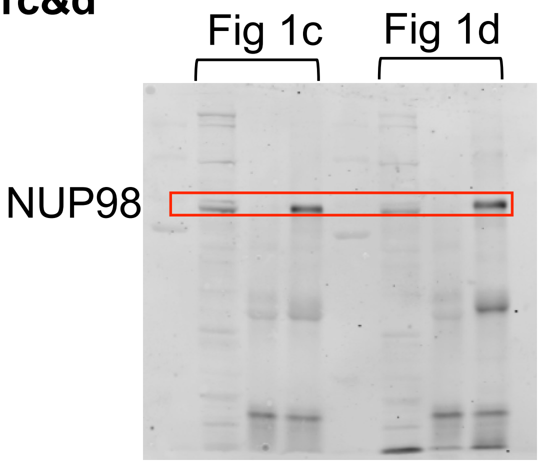

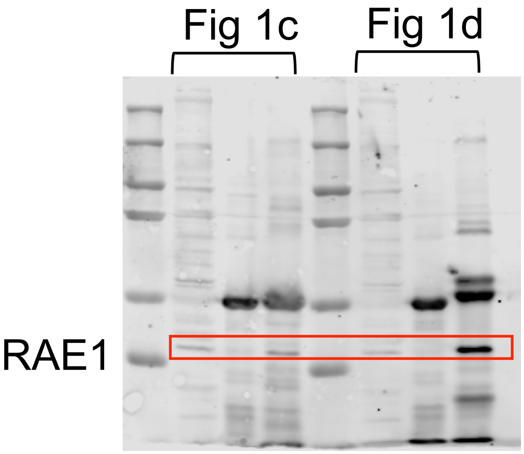

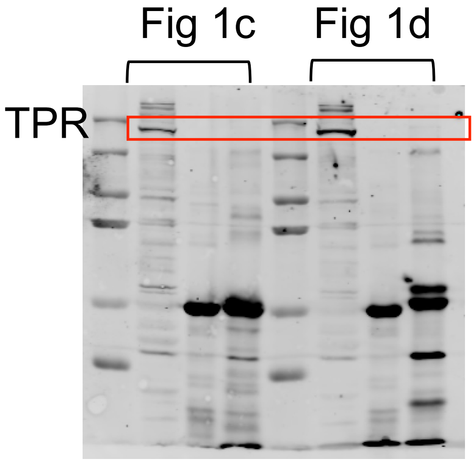

Fig 1f

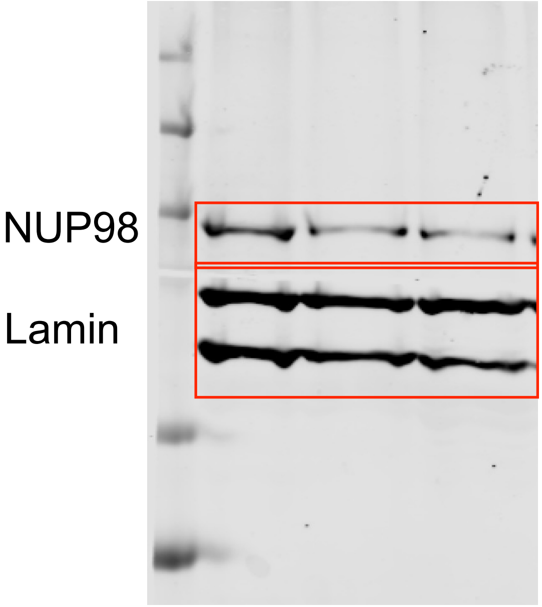

Supplementary Figure 9. (continued)

Fig 1i

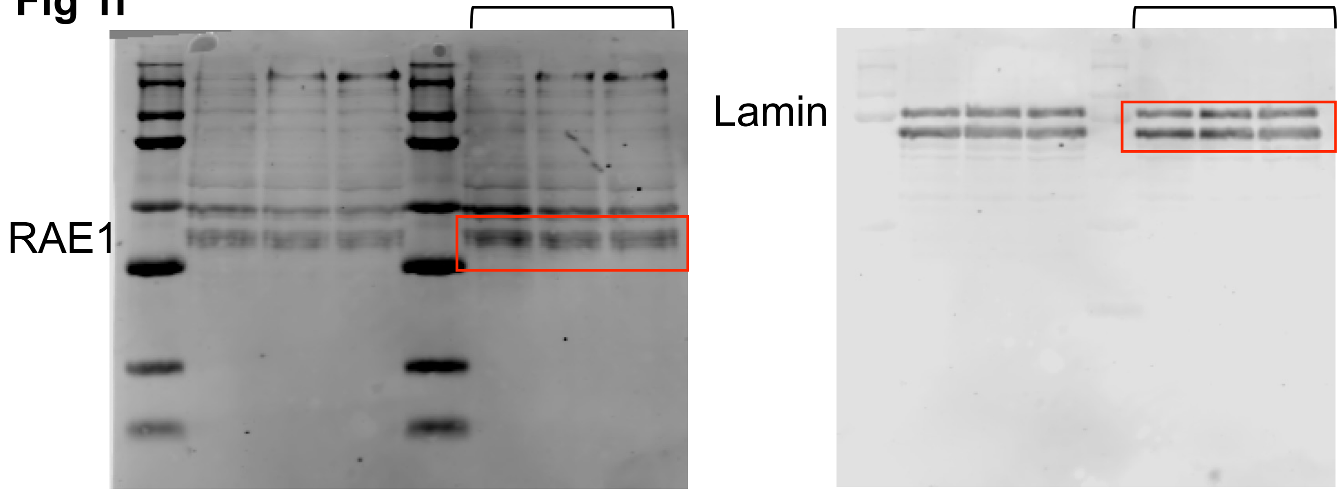

Fig 3p

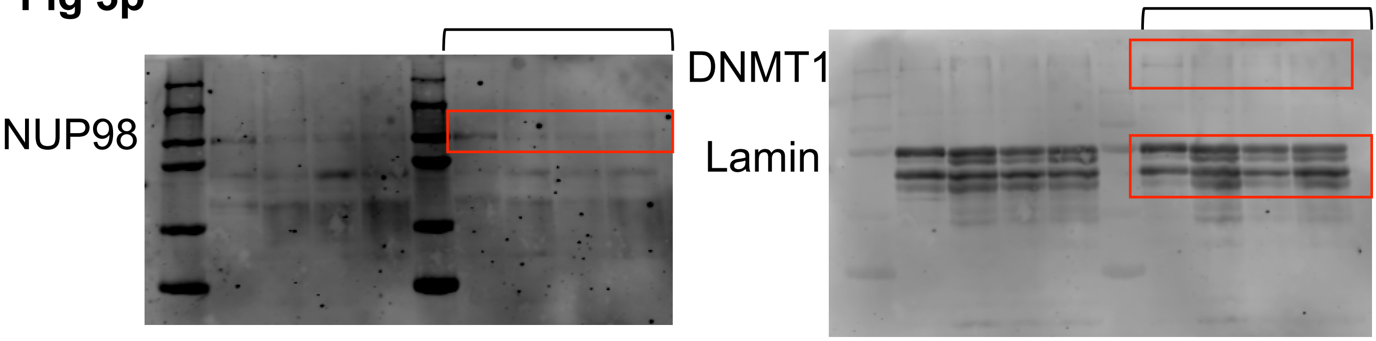

Fig 3r

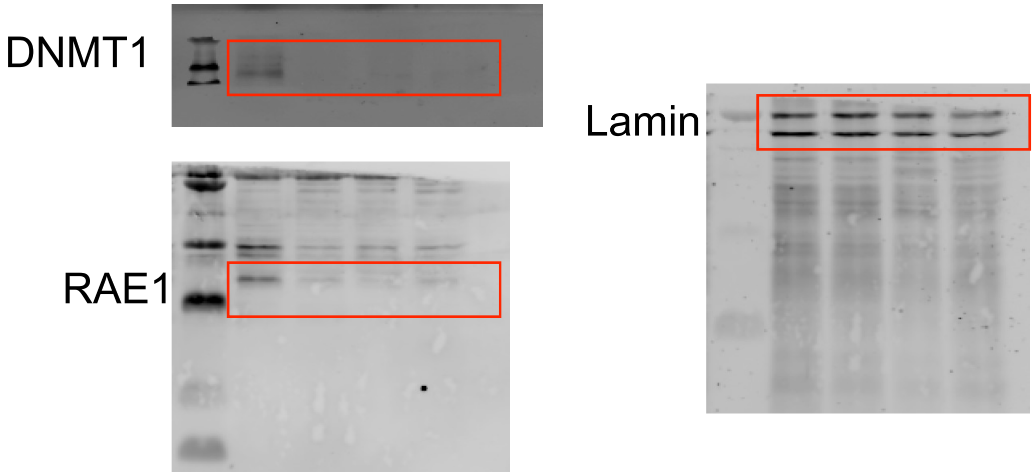

Supplementary Figure 9. (continued)

Fig 4e

NUP98

HDAC1

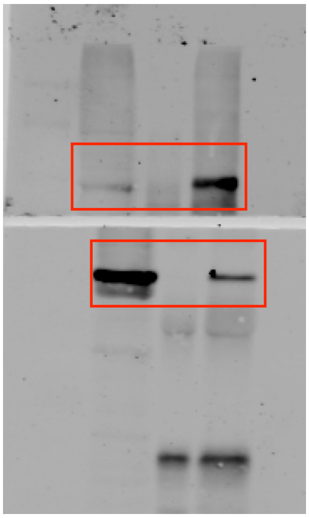

Fig 4f

HDAC1

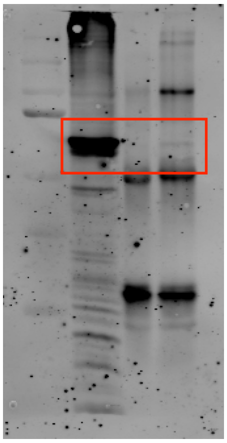

RAE1

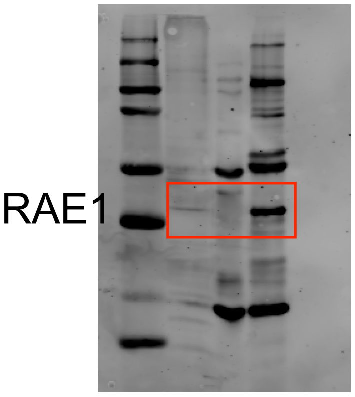

Fig S1a

NUP98

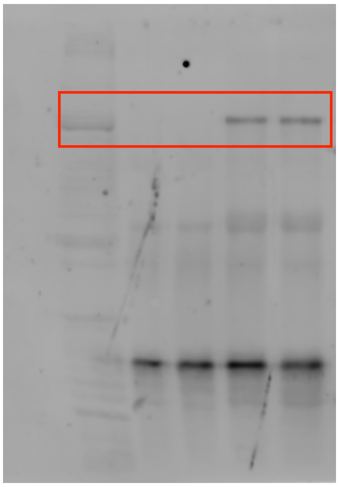

RAE1

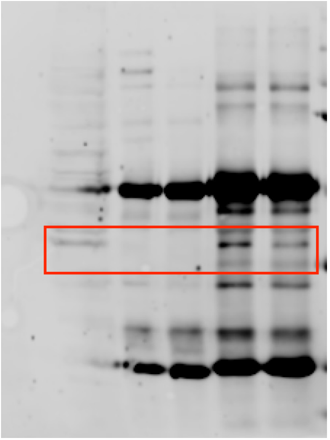

Supplementary Figure 9. (continued)

Fig S2a

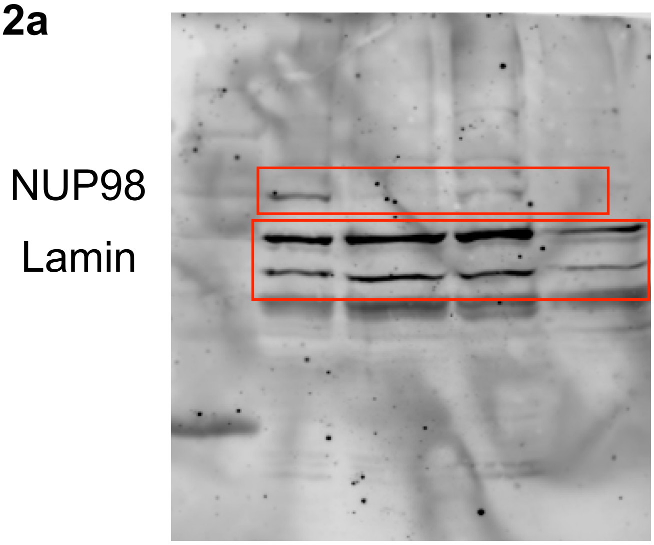

Fig S2c

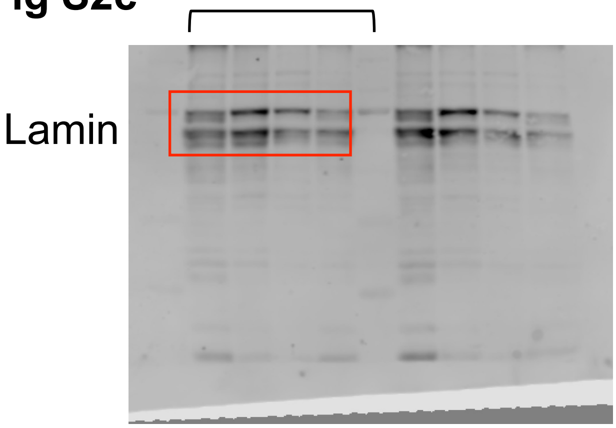

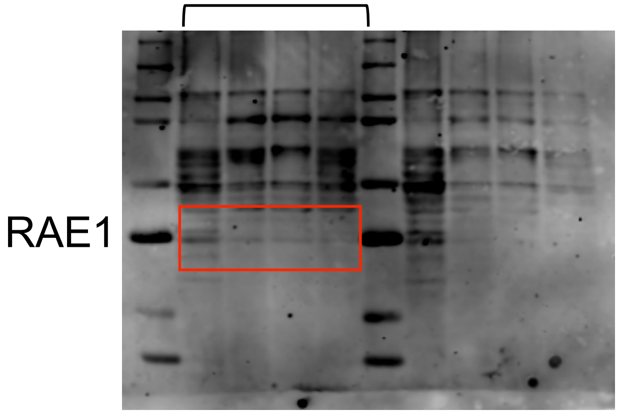

Fig S3a

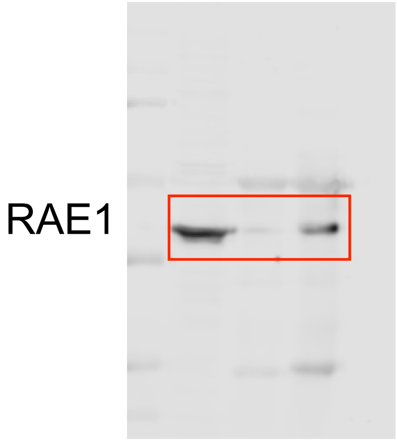

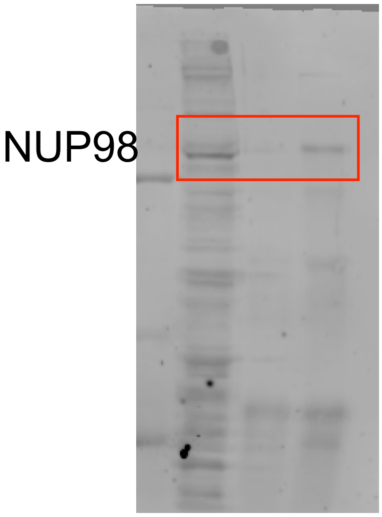

Supplementary Figure 9 (continued)

Fig S4a

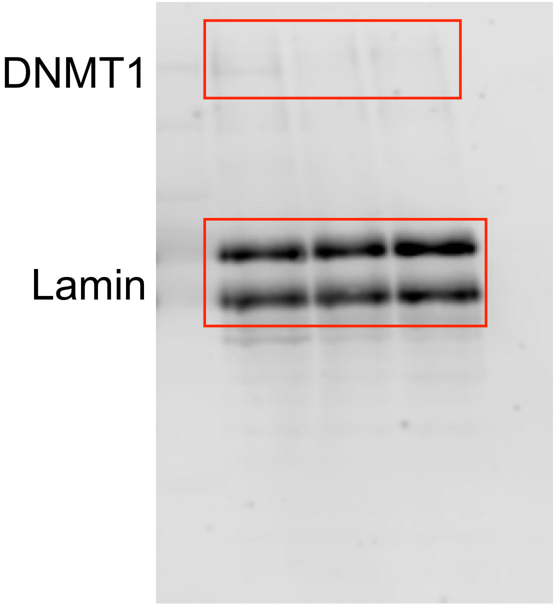

Fig S5c

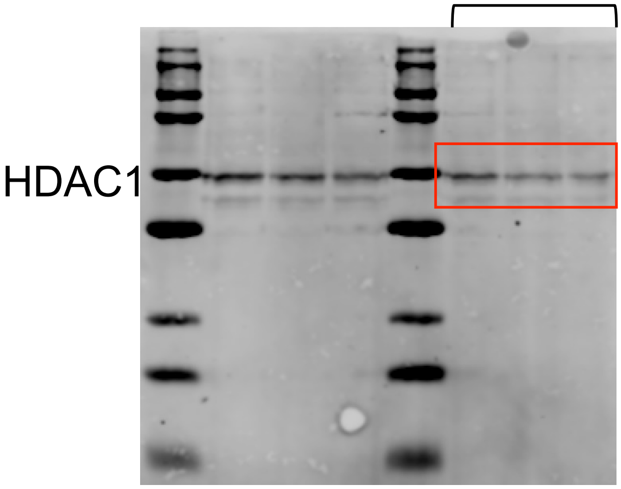

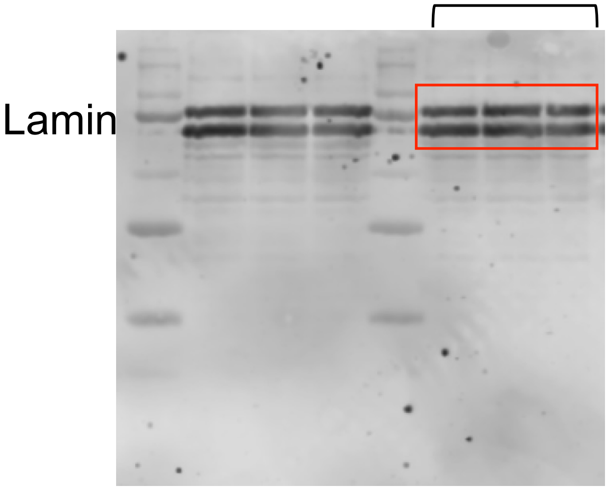

Supplement: Supplementary file 2 — Supplementary Information [file 42003_2023_5043_MOESM2_ESM.pdf]
