# Supplementary material for: NUP98 and RAE1 sustain progenitor function through HDAC-dependent chromatin targeting to escape from nucleolar localization
Source: Commun Biol. 2023 Jun 23;6:664. doi: 10.1038/s42003-023-05043-2 (PMC10290086; doi:10.1038/s42003-023-05043-2)
Supplement: Supplementary file 3 — Description of Additional Supplementary Files [file 42003_2023_5043_MOESM3_ESM.pdf]

Description of Additional Supplementary Files

File name: Supplementary Data 1
Description: Differentially Expressed Genes with NUP98 knockdown

File name: Supplementary Data 2
Description: Differentially Expressed Genes with RAE1 or NUP98 knockdown

File name: Supplementary Data 3
Description: NUP98_ChIP_peaks_UD(1554)

File name: Supplementary Data 4
Description: Co-occupancy at NUP98 ChIP-seq peaks by other marks/regulators in human primary keratinocytes

File name: Supplementary Data 5
Description: NUP98 ChIP direct target genes (101).

File name: Supplementary Data 6
Description: Differentially Expressed Genes with HDAC inhibition.

File name: Supplementary Data 7
Description: List of Oligos Used in This Study

File name: Supplementary Data 8
Description: Source Data behind the graphs in this manuscript
