# Supplementary material for: NUP98 and RAE1 sustain progenitor function through HDAC-dependent chromatin targeting to escape from nucleolar localization
Source: Commun Biol. 2023 Jun 23;6:664. doi: 10.1038/s42003-023-05043-2 (PMC10290086; doi:10.1038/s42003-023-05043-2)
Supplement: Supplementary file 6 — Reporting Summary [file 42003_2023_5043_MOESM6_ESM.pdf]

nature portfolio

Corresponding author(s): Xiaomin Bao

Last updated by author(s): Jun 10, 2023

Reporting Summary

Nature Portfolio wishes to improve the reproducibility of the work that we publish. This form provides structure for consistency and transparency in reporting. For further information on Nature Portfolio policies, see our Editorial Policies and the Editorial Policy Checklist.

Statistics

For all statistical analyses, confirm that the following items are present in the figure legend, table legend, main text, or Methods section.

n/a	Confirmed	
☐	☒	The exact sample size (n) for each experimental group/condition, given as a discrete number and unit of measurement
☐	☒	A statement on whether measurements were taken from distinct samples or whether the same sample was measured repeatedly
☐	☒	The statistical test(s) used AND whether they are one- or two-sided<br>*Only common tests should be described solely by name; describe more complex techniques in the Methods section.*
☐	☒	A description of all covariates tested
☐	☒	A description of any assumptions or corrections, such as tests of normality and adjustment for multiple comparisons
☐	☒	A full description of the statistical parameters including central tendency (e.g. means) or other basic estimates (e.g. regression coefficient) AND variation (e.g. standard deviation) or associated estimates of uncertainty (e.g. confidence intervals)
☐	☒	For null hypothesis testing, the test statistic (e.g. F, t, r) with confidence intervals, effect sizes, degrees of freedom and P value noted<br>*Give P values as exact values whenever suitable.*
☒	☐	For Bayesian analysis, information on the choice of priors and Markov chain Monte Carlo settings
☒	☐	For hierarchical and complex designs, identification of the appropriate level for tests and full reporting of outcomes
☒	☐	Estimates of effect sizes (e.g. Cohen's d, Pearson's r), indicating how they were calculated

Our web collection on statistics for biologists contains articles on many of the points above.

Software and code

Policy information about availability of computer code

Data collection	The qRT-PCR data were acquired using QuantStudio Design & Analysis Software 1.3.1 (Thermo Fisher)<br>Western blots images were collected using Image studio software version 5.2 (LI-COR)<br>Immunofluorescence images were collected using the EVOS FL Auto 2 Imaging system software (Thermo Fisher)
Data analysis	This paper does not report original code.<br>Quantification of western blots was done using Image studio software version 5.2 (LI-COR).<br>Immunofluorescence images were analyzed using ImageJ version 2.1.0<br>Statistical analysis for qPCR, Western, and Immunofluorescence was done using GraphPad Prism version 9.<br>For RNA and ChIP-seq the software is described in the Methods section and R Studio version 3.5.2 was used for analysis. Other softwares used include bedtools, EdgeR, Diffbind, Samtools, DESeq2, Homer, and DAVID Gene Ontology analyses.

For manuscripts utilizing custom algorithms or software that are central to the research but not yet described in published literature, software must be made available to editors and reviewers. We strongly encourage code deposition in a community repository (e.g. GitHub). See the Nature Portfolio guidelines for submitting code & software for further information.

Data

Policy information about availability of data

All manuscripts must include a data availability statement. This statement should provide the following information, where applicable:

- Accession codes, unique identifiers, or web links for publicly available datasets
- A description of any restrictions on data availability
- For clinical datasets or third party data, please ensure that the statement adheres to our policy

The RNA-seq and ChIP-seq data have been deposited to GEO, with the accession number #GSE150799 (including both GSM6797237-GSM6797258). The reviewer token is crurwkispvmbjcv.

Human research participants

Policy information about studies involving human research participants and Sex and Gender in Research.

Reporting on sex and gender	Keratinocytes were isolated from surgically discard skin specimen from de-identified donors. Keratinocytes isolated from 6 donors were pooled for all the experiments performed in this study. Therefore, this study is not focused on the characterization of cells from individual donors.
Population characteristics	All the experiments were performed using pooled primary keratinocytes pooled from 6 de-identified donors, at early passages. The purity of each batch of primary keratinocytes were confirmed based on cell morphology using microscopy.
Recruitment	Not applicable
Ethics oversight	This research was reviewed by Northwestern University Institute Review Board (IRB) and assigned a determination of NOT HUMAN RESEARCH.

Note that full information on the approval of the study protocol must also be provided in the manuscript.

Field-specific reporting

Please select the one below that is the best fit for your research. If you are not sure, read the appropriate sections before making your selection.

☒ Life sciences ☐ Behavioural & social sciences ☐ Ecological, evolutionary & environmental sciences

For a reference copy of the document with all sections, see nature.com/documents/nr-reporting-summary-flat.pdf

Life sciences study design

All studies must disclose on these points even when the disclosure is negative.

Sample size	Primary human keratinocytes were pooled from at least six donors, as is standard for primary keratinocyte culture (Bao et al, 2017). Gene knockdown experiments were done using two non- targeting controls and two or three independent shRNA targeting the gene of interest as was done in Bao et al 2017. ChIP experiments were completed with at least two biological replicates (Lloyd et al, 2022). RT-qPCR experiments were all done with at least three biological replicates and each biological replicate was done in technical triplicates. RNA-seq data was also done in biological duplicate or triplicates. For tissue regeneration assay, at least two organotypic cultures were preprepared for each sample (Li et al, 2021). All immunofluorescence data were done in biological triplicates and a minimum of three images were used for quantification between replicates. All replicates showed the same trend; one representative image was shown.
Data exclusions	No data were excluded from analysis.
Replication	All experiments were performed in either technical or biological replicates. All replicates generated were used for this paper.
Randomization	Six independent (de identified) donors were used for pooling primary human keratinocytes for all experiments.
Blinding	Blinding was not required for this study.

Reporting for specific materials, systems and methods

We require information from authors about some types of materials, experimental systems and methods used in many studies. Here, indicate whether each material, system or method listed is relevant to your study. If you are not sure if a list item applies to your research, read the appropriate section before selecting a response.

Materials & experimental systems

n/a	Involved in the study
☐	☒ Antibodies
☐	☒ Eukaryotic cell lines
☒	☐ Palaeontology and archaeology
☒	☐ Animals and other organisms
☒	☐ Clinical data
☒	☐ Dual use research of concern

Methods

n/a	Involved in the study
☐	☒ ChIP-seq
☒	☐ Flow cytometry
☒	☐ MRI-based neuroimaging

Antibodies

Antibodies used

Antibodies used for western in this study include NUP98 C-7 (Santa Cruz Biotechnology, sc-74553), Lamin A/C (Santa Cruz Biotechnology, sc-376248), RAE1 (Santa Cruz Biotechnology, sc-393252, TPR (Bethyl, A300-828A), NUP153 (Bethyl A301-788A-M), DNMT1 (cell signaling, D63A6), and HDAC1 (cell signaling, D5C6U).
The antibodies used for ChIP: NUP98 C-7 (Santa Cruz Biotechnology, sc-74553), HDAC1 (Diagenode, C15410325), HA (66006, proteintech.

Validation

NUP98 C-7 (Santa Cruz Biotechnology, sc-74553) was validated for WB, IP, IF and ELISA by manufacturer. This antibody was been cited 7 times. The information can be found at: https://www.scbt.com/p/nup98-antibody-c-7.

Lamin A/C (Santa Cruz Biotechnology, sc-376248) was validated for WB, IP, IF, IHC(P) and ELISA by manufacturer. This antibody was been cited 206 times. The information can be found at: https://www.scbt.com/p/lamin-a-c-antibody-e-1.

Mrnp1 (aka: RAE1) (Santa Cruz Biotechnology, sc-393252) was validated for WB, IP, IF, IHC(P) and ELISA by manufacturer. This antibody was been cited 2 times. The information can be found at: https://www.scbt.com/p/mrnp41-antibody-h-3

TPR (Bethyl, A300-828A) was validated for WB, IP, IF, IHC(P) by manufacturer. The information can be found at: https://www.thermofisher.com/antibody/product/TPR-Antibody-Polyclonal/A300-828A

NUP153 (Bethyl A301-788A-M) was validated for WB, IF, and IP by manufacturer. The information can be found at: https://www.thermofisher.com/antibody/product/NUP153-Antibody-Polyclonal/A301-788A.

DNMT1 (cell signaling, D63A6, mAb #5032) was validated for WB, IHC, and IF by manufacturer. This antibody was been cited 151 times. The information can be found at: https://www.cellsignal.com/products/primary-antibodies/dnmt1-d63a6-xp-rabbit-mab/5032

HDAC1 (cell signaling, D5C6U, mAb #34589) was validated for WB, IP, IF, and ChIP by manufacturer. This antibody was been cited 88 times. The information can be found at: https://www.cellsignal.com/products/primary-antibodies/hdac1-d5c6u-xp-rabbit-mab/34589

HDAC1 (Diagenode, C15410325) was validated for ChIP, ELISA, WB, and IF by manufacturer. This antibody was been cited 16 times. The information can be found at: https://www.diagenode.com/en/p/hdac1-polyclonal-antibody-premium-50-ug

HA (66006, proteintech, CloneNo 1F5C6) was validated for WB, IP, IF and FC by manufacturer. This antibody was been cited 133 times. The information can be found at: https://www.ptglab.com

Eukaryotic cell lines

Policy information about cell lines and Sex and Gender in Research

Cell line source(s)

Primary human keratinocytes were used for all experiments. Cell lines were not used for experimental data or conclusions in this study. 293T or phoenix cells were used for lenti/retro virus production to infect primary human keratinocytes with trans gene. Clonogenicity assays were done using 3T3 cells as feeder cells. These cell lines were generous gifts from the Khavari Lab at Stanford University. The cell lines are also available commercially from ATCC (CRL-3216, CRL-1658, CRL-3213).

Authentication

Cell lines were authenticated by morphology.

Mycoplasma contamination

The "MycoFLuor Mycoplasma Detection Kit" (cat#M7006) was used to test all cell lines and primary human keratinocytes. The results for all tested cell lines was negative for mycoplasma.

Commonly misidentified lines
(See ICLAC register)

No commonly misidentified cell lines were used in this study.

ChIP-seq

Data deposition

☒ Confirm that both raw and final processed data have been deposited in a public database such as GEO.

☒ Confirm that you have deposited or provided access to graph files (e.g. BED files) for the called peaks.

Data access links<br>*May remain private before publication.*	accession number #GSE150799 (including both GSM6797237-GSM6797258). The reviewer token is crurwkispvmbjcv.
Files in database submission	HDAC1-ChIPseq_UDKC_rep1 [file name= HDAC1_set1_UD]<br>HDAC1-ChIPseq_UDKC_rep2 [file name= HDAC1_set1_UD]<br>HDAC1_ChIPseq_DFKC_rep1 [file name= HDAC1_set1_DF]<br>HDAC1_ChIPseq_DFKC_rep2 [file name= HDAC1_set2_DF]<br><br>NUP98-ChIPseq_set1_UDKC [file name= NUP98_ChIP-seq_keratinocytes_set1_UD]<br>NUP98-ChIPseq_set1_DFKC [file name= NUP98_ChIP-seq_keratinocytes_set1_DF]<br><br>NUP98-ChIPseq_set2_UDKC [file name= NUP98_ChIP-seq_keratinocytes_set2_UD]<br>NUP98-ChIPseq_set2_DFKC [file name= NUP98_ChIP-seq_keratinocytes_set2_DF]<br>NUP98-ChIPseq_UDKC_rep3 [file name= NUP98-ChIPseq_UDKC_rep3]<br>NUP98-ChIPseq_UD KC_(DMSO control) [file name= NUP98-ChIP_DMSO]<br>NUP98-ChIP_HDACinhibition#1 (ROM) [file name= NUP98-ChIP_ROM]<br>NUP98-ChIP_HDACinhibition#2 (SAHA) [file name= NUP98-ChIP_SAHA]<br><br>HA-RAE1 ChIP-seq rep1 [file name=HA-RAE1]<br>HA-RAE1 ChIP-seq rep2 [file name=HA-RAE1_Rep2]<br><br>HDAC1_ChIP-seq_UDKC_rep3 [file name = HDAC1-ChIP-seq_UDKC]<br>HDAC1_ChIP-seq_UDKC_rep4 [file name= HDAC1-ChIP-seq_UDKC_rep2]
Genome browser session<br>(e.g. UCSC)	no longer applicable

Methodology

Replicates	All ChIP-seq experiments were preformed with 2 biological replicates.
Sequencing depth	~20 million reads passed MAPQ score of 30 for all libraries
Antibodies	The antibodies used for ChIP: NUP98 C-7 (Santa Cruz Biotechnology, sc-74553), HDAC1 (Diagenode, C15410325), HA (66006, proteintech).
Peak calling parameters	ChIP-seq peaks were called using MACS2 v2.2.7.1 with q value < 0.01 and narrow peak calling.
Data quality	MAPQ score minimum of 30. q value of 0.01 for calling peaks
Software	ChIP-seq reads were mapped to GRCh38 (NCBI) using BWA-MEM v0.7.17 with default parameters. MACS2 was used for peak calling
